# Snake markings facilitate diverse anti-predator functions depending on habitat and viewing angle

**DOI:** 10.1093/beheco/arag051

**Published:** 2026-05-18

**Authors:** Zixiang Li, George R A Hancock, Laura A Kelley, Martin Stevens

**Affiliations:** Centre for Ecology and Conservation, University of Exeter, Penryn TR10 9FE, United Kingdom; Centre for Ecology and Conservation, University of Exeter, Penryn TR10 9FE, United Kingdom; Centre for Ecology and Conservation, University of Exeter, Penryn TR10 9FE, United Kingdom; Centre for Ecology and Conservation, University of Exeter, Penryn TR10 9FE, United Kingdom

**Keywords:** camouflage, snake pattern, aposematism, defensive coloration, distance, angle, zigzag, local adaptation

## Abstract

Animals employ diverse coloration strategies to reduce predation risk, with patterns playing a key role in camouflage or for signaling unprofitability. Snakes exhibit various markings, but the specific antipredator functions of these patterns remain debated. We investigated how different snake patterns interact with habitat background and predator viewing conditions to influence camouflage. Using artificial snake models with 4 patterns (uniform, stripe, spot, zigzag) and 2 body colors (brown, gray), we assessed detectability across different natural habitats in southwest UK. We used visual modeling to simulate mammalian and avian predators and quantified background matching and edge disruption across varying visual distance and angles. Our results show that patterns affect camouflage depending on habitat and observer perspective. Spot and zigzag patterns increased edge disruption, particularly at greater distances, while striped patterns provided less effective concealment. However, zigzag patterns increased the contrast of snakes with conspicuous coloration in open habitats, suggesting potential aposematism. Viewing angle significantly influenced camouflage, with higher angles breaking edge disruption of zigzag and spot patterns, which also decreases as an observer approaches. Our findings highlight the multifunctionality of varied snake patterns, demonstrating their ability to facilitate both crypsis and aposematism depending on the ecological context. These results emphasize the importance of integrating habitat, body color, and predator perspective in understanding antipredator adaptations.

## Teaser

Many snakes have bold color patterns, but how do habitat and viewing conditions change their functions? We found that zigzag and spot patterns can disrupt the snake's outline, reducing the likelihood of detection. However, for brightly colored snakes, the zigzag pattern can enhance their conspicuousness in open habitats, indicating a warning function. At higher observation angles, it appears that patterns change from hiding the snake to warning observers of their presence.

## Introduction

Predator–prey interactions typically begin with the detection and recognition of prey (Endler 1978; Galloway et al. 2020). Animal coloration involves various defensive strategies that cover a spectrum from inhibiting detection or recognition to promoting visibility and identification (Ruxton et al. 2018). Cryptic coloration minimizes the signal-to-noise ratio of prey against the background (Merilaita et al. 2017; Michalis et al. 2017) and exploits specific visual processes in predators (Stevens et al. 2006; Troscianko et al. 2009; Cuthill 2019). By contrast, aposematic signals tend to operate by strengthening visual signals associated with unprofitable traits, which encourage avoidance learning and innate avoidance (Cott 1940; Sherratt and Beatty 2003; Stevens and Ruxton 2012).

Anti-predator coloration is mediated not only by color but also by patterning, as the spatial arrangement of markings can alter detectability and recognition (Cott 1940, Troscianko et al. 2018). In addition to blending in with the general environment (background matching), camouflage patterns can impair detection or recognition by obscuring an object's outline and creating false but relatively high contrast edges, masking true edge information of the body through disruptive coloration (Thayer 1909; Cott 1940; Cuthill et al. 2005; Troscianko et al. 2009). Adjacent patterns may also show strong contrasts, and provide false depth cues, further hiding an object (Stevens et al. 2009; Kelley et al. 2022). Alternatively, pattern evolution may instead favor higher conspicuousness and markings that highlight body edges, such as in the use of warning signals to avoid predation (Lindstedt et al. 2008; Stevens and Ruxton 2012), particularly if concealment is constrained by large body size (Smith et al. 2016; Pembury Smith and Ruxton 2021; Yu et al. 2024) or developmental constraints (Cuthill et al. 2006).

In reality, camouflage and conspicuousness are ends of a spectrum and the above strategies are not always mutually exclusive. For example, there is good evidence that the same patterns can function in both camouflage and as warning signals depending on viewing distance (eg Barnett and Cuthill 2014). Furthermore, in heterogeneous habitats, the background usually varies in time and space, which affects signal detectability and the efficacy of camouflage, and animals may be observed against different backgrounds (Endler 1993; Merilaita and Dimitrova 2014). This raises an under-explored issue, as to whether the same external pattern might support different functions across environments and predator viewing conditions. For example, the efficacy of different anti-predator defenses may be influenced by how far away a predator is from a prey item, and the angle at which it is observed. Although the mechanisms of defensive coloration are well characterized, it remains unclear when the same external pattern can serve different functions across environments and predator viewing conditions.

Snakes provide an excellent system to address the above issues since they occupy wide geographic ranges spanning diverse habitats, and they frequently travel between different patches, encountering various substrates (Andrén 1985; Shine and Madsen 1994). Phylogeographic studies linked variation in snake patterns to habitat types (Allen et al. 2013; Santos et al. 2014, 2018; Pizzigalli et al. 2020), indicating a relationship with cryptic effectiveness. Snakes exhibit a rich variety of patterns, from stripes (eg ladder snake *Rhinechis scalaris*) and spots (eg smooth snake *Coronella austriaca*), to the iconic zigzags of many viper species (Allen et al. 2013). Zigzag-like dorsal motifs are most characteristic of vipers, but similar vertebral zigzags also occur in some non-viper snakes (eg some species of natricine snakes such as *Natrix maura*, uropeltid snakes such as *Rhinophis zigzag*, and cat-eyed snakes such as *Leptodeira ornata*). The body color of many snakes is typically grayish and brown, although some species rely on bright aposematic coloration. Banded and striped patterns have been proposed to serve motion dazzle or flicker-fusion functions, blurring into uniform colors during movement (Brodie 1992; Allen et al. 2013; Titcomb et al. 2014). Zigzag patterns have been considered both as disruptive coloration (Cott 1940; Shine and Madsen 1994), with cryptic effects (Santos et al. 2018; Valkonen et al. 2020), and as warning signals against avian attacks (Santos et al. 2018; Valkonen et al. 2020); they may also function in a similar manner to striped patterns by producing motion dazzle and flicker fusion effects to mammalian predators, which have a lower critical flicker fusion frequency than birds (Valkonen et al. 2020).

Snakes face threats from avian predators many with likely tetrachromatic, high-acuity vision (Cuthill et al. 2006; Martin 2022), as well as mammalian predators (eg badgers and foxes) with dichromatic, lower-acuity vision (Jacobs 2009). Camouflage effectiveness can vary significantly across these visual systems (Isaac and Gregory 2013; Kang et al. 2016; Goedert et al. 2021), and given that snake patterns are centered on the dorsal surface, the perceived boundaries of such 3D objects will vary with viewing angle (Stevens and Merilaita 2009). Moreover, a predator's foraging habits and viewing angle may also influence the maximum possible detectable distance, which may drive shift in pattern function from cryptic to aposematic as the predator approaches due to distance-dependence of conspicuousness (Tullberg et al. 2005; Bohlin et al. 2008; Barnett and Cuthill 2014; Barnett et al. 2018). Although prior studies have addressed the anti-predator function of snake coloration, how these patterns and their function interact with habitat, body color, and viewing angle, and consequences for detectability remain insufficiently tested. We address this general question using snake patterns broadly, focusing on a few common general forms (eg spots, stripes, zigzags), because previous studies have offered differing interpretations of the function and underlying mechanisms.

To test how snake body color and pattern influence camouflage effectiveness, and how this varies with background type, predator visual system and viewing angle, we assessed the detectability of artificial snake models across contrasting visual contexts. Our approach was designed to capture variation in coloration and patterning ie broadly representative of many snake species, though the specific ecological context of our experimental setup is especially relevant to vipers, which can utilize varied markings across habitats and which have been a focus of much past research (see above). We photographed brown and gray artificial snake models with common snake patterns in various snake habitats in the southwest UK from different heights, then modeled camouflage effectiveness based on the visual systems of dichromatic (red fox, *Vulpes vulpes*) (Malkemper and Peichl 2018) and tetrachromatic predators (black kite, *Milvus migrans*) (Potier et al. 2016). We predicted that zigzag and spot patterns would enhance camouflage in both avian and mammalian visual systems, but the camouflage efficacy of different patterns would vary depending on habitat types. By contrast, the stripe pattern would not enhance or decrease conspicuousness, because these patterns require movement for effective camouflage. Additionally, the elongated body shape of snakes and the uneven distribution of patterns suggests that the visible pattern and its camouflage effectiveness might vary with viewing angle, and that viewing distance would also affect camouflage.

## Materials and methods

### Artificial model preparation and research sites

**Figure 1:**
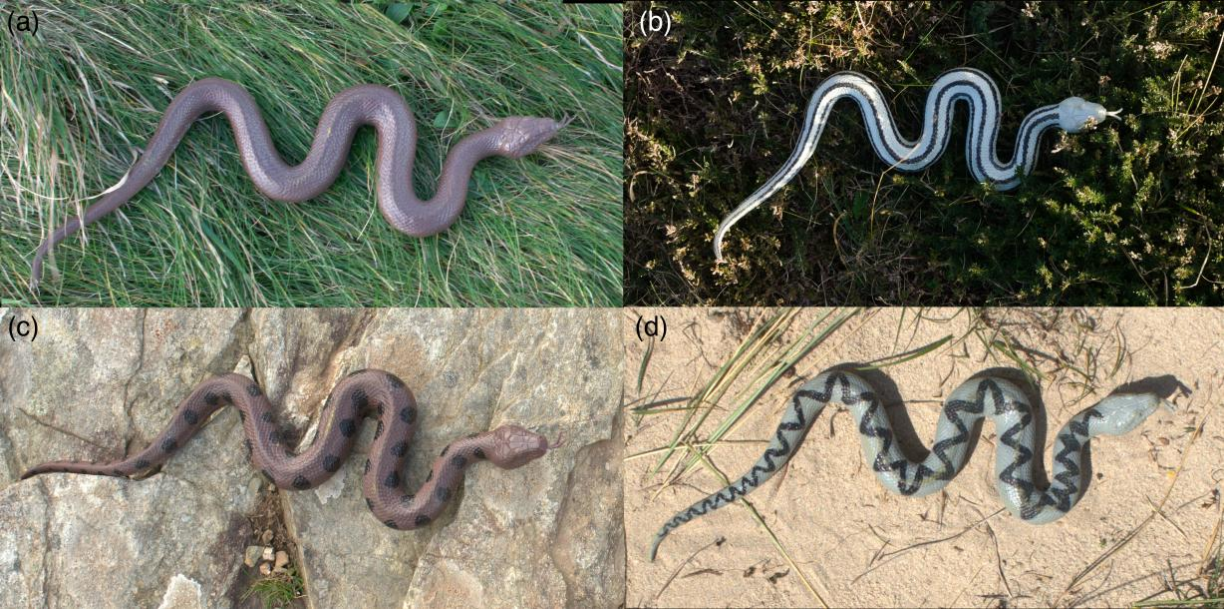
Representative snake model treatments used to test the effect of coloration and patterns in different habitats: a) uniform snake in meadow habitat; b) striped snake in heathland; c) spotted snake in rocky background; d) zigzag snake in sand habitat.

Replicas of snakes ∼25 cm in length and 2.5 cm in diameter were created using soft rubber snake models (Cobee). We selected brown and gray as 2 representative color morphs commonly reported for a variety of patterned snakes, and the pattern types we chose are prevalent across many snake taxa, such that our treatments were chosen to represent general snake color variation. We painted 32 rubber snakes (4 models for each color-pattern group) using spray paint in brown (151 Products Ltd, brown) and gray (Paint Factory Ltd, light gray). Four distinct pattern types were hand-painted in black paint (Edding) on these replicas, the size approximately estimated according to photos of real snakes: uniform color (U), a solid base color without any pattern, serving as the control group; stripe pattern (L), featuring 3 parallel stripes running along the body axis, with stripes ∼0.5 cm wide; spot pattern (S), consisting of 2 rows of paired spots on the lateral body, each spot 1.5 ± 0.2 cm in diameter, getting smaller when closer to the narrower tail; and zigzag pattern (Z), a dorsal zigzag design, with width of ∼0.8 cm (Fig. 1). The stripe patterns are unlikely to disrupt the snake's outline regardless of viewing angle because the markings do not intersect the body margins, and represent a class of striped snakes such as ladder snake and grass snake (*Natrix natrix*), and also provide a baseline for the effects of black paint and contrast. The spotty and zigzag pattern are representative of species such as adders (*Vipera berus*). The spots should be fully visible from lower angles but less so from an overhead perspective. The zigzag pattern is primarily central on the dorsal side and may disrupt the snake's outline when viewed from the side.

After painting, we photographed the models to calculate the black pattern coverage percentage on the body, using a Sony A7 camera with a 28 to 70 mm lens, mounted on a tripod 1 m above the replica. We quantified the pattern areas using ImageJ 1.53v, ensuring that the percentage differences among different types of patterns in a vertical view were confined to <10%. Given that small differences in apparent coverage can also vary with viewing angle and occlusion, the relatively small differences in pattern coverage between models is unlikely to affect our results.

We conducted the study within the nature reserves of Godrevy and Lizard in Cornwall, United Kingdom (Fig. S1). Given the widespread distribution of snakes across Europe, their habitats encompass various landscapes. We used 4 primary habitat types: (i) Meadows—open fields dominated by diverse herbaceous species and graminoids; (ii) Heathlands—low shrublands dominated by low-stature ericaceous shrubs; (iii) Rocky Slopes—semi-open areas with sparse vegetation; and (iv) Sands—semi-open yet generally brighter landscapes (Fig. 1). The 2 locations are predominantly meadow and heath, but encapsulate all 4 habitat types.

At each reserve, we followed a transect which spanned all 4 habitat types for the placement of snake models. Along each transect, for each habitat type, we haphazardly selected 20 points to place a snake model, giving 80 points where a snake model was placed per reserve (160 points in total over 2 reserves, 40 points per habitat type). We positioned snake models 4 m either side of the transect, ensuring a 30-m minimum distance between points within the same habitat type to provide representative habitat samples. At each chosen point, every snake color-pattern treatment (2 color × 4 pattern) was placed on the ground and photographed, giving a total of 1,280 specific snake model-background replicates (160 points of snake photography × 8 snake color-pattern treatments). Photographs were taken at different angles (see below), resulting in 2,560 photographs in total. Weather and light conditions during photography sessions were recorded. The University of Exeter Animal Ethics Committee approved all procedures (Application ID 528352).

### Photography and image analysis

At each point, we selected the snake models haphazardly by blindly choosing a model from a bag. The snakes were directly placed on the ground at the chosen point of selected habitat, and the position of the snake was adjusted to minimize obstruction from vegetation. For each point, we photographed a snake model of each of the 4 pattern kinds, in both body colors, and from 2 angles. For photographing the snake models, a Sony A7 digital camera with a 28 to 70 mm lens was used, positioned ∼1.5 m from the subject under natural light. Manual adjustments were made to the camera settings for white balance, setting the aperture at f/6.3 and ISO at 160. Each photograph included a DIGITAL HD MENNON gray (18%) card and a ruler (Stevens et al. 2007; Troscianko and Stevens 2015) for calibration of the images. Each model snake was photographed twice; from directly overhead, and at a 20 to 30° oblique angle, as estimated by an inclinometer. The capture format was RAW for all images. The snake model was returned to the bag after photography and models mixed evenly.

All image processing and analysis were performed using the Multispectral Image Calibration and Analysis Toolbox v2.2.2 (Troscianko and Stevens 2015), in ImageJ 1.53v (Schneider et al. 2012). Pattern analysis used the “Batch Multispectral Analysis” tool, while color and pattern analysis calculations were conducted using the “Batch RNL Chromaticity” tool (Troscianko et al. 2016). Our replicas and paints did not allow for control of ultraviolet (UV) reflection and our imaging protocol did not capture UV; consequently, analyses were restricted to the visible-range (400 to 700 nm) (see Discussion).

The images were calibrated using the gray standard to normalize the images and standardize across light conditions and were saved as 32-bit multispectral images in.tiff format. Subsequently, a scale bar was added in accordance with the ruler in each photo. The snake model outline in each image was manually selected as the region of interest (ROI), carefully excluding background obstructions that interfered with the snake outline, unless they completely obscured it. A fitted rectangle encompassing the snake ROI defined the local background, while a bounding circle determined the global background for habitat metric calculations.

#### Pattern matching

We modeled pattern appearance through the visual system types of 2 putative predators of snakes: the dichromatic mammal red fox and the tetrachromatic avian predator black kite. Due to the unavailability of specific spectral receptor sensitivity data for these animals, we used the visual systems of domestic dogs (*Canis familiaris*) and the peafowl (*Pavo cristatus*) as proxies, assuming similar sensitivities, as is common in many studies (Perez-Martinez et al. 2020; Nokelainen et al. 2021, 2024). The photoreceptor sensitivities of dog (Neitz et al. 1989) and peafowl (Hart 2001) were included in the mica toolbox (Troscianko and Stevens 2015). For the peafowl visual system, we excluded the violet sensitive cone as the snake replicas lacked UV reflection and UV reflectance in the background was very low. The luminance channel for peafowl was focused on the middle to long wave spectrum which is mediated by double cones, while for domestic dogs, it was centered on the long/medium (L/M-) wavelength-sensitive cones with peak spectral sensitivity of 555 nm.

To analyze how closely the different snake pattern types match their background environments, we utilized a granularity analysis, a technique based on Fourier analysis and bandpass filtering. This method has been previously employed in studies of cuttlefish, shore crabs, rockpool fish, and egg pattern analysis (Chiao et al. 2007; Smithers et al. 2017; Stevens et al. 2017; Price et al. 2019). The bandpass filter range was set to 30 octaves, starting from 1 up to 25 mm (matching the length of the replica), with each step increasing by a factor of 1.2. This range was chosen to measure pattern differences. In our analysis, we prioritized achromatic information over color, as it usually provides more insights into spatial pattern details (Olsson et al. 2018). The standard deviation of luminance across each spatial scale was calculated to represent the energy at that scale.

Pattern energy difference (PED) was used to quantify pattern matching. PED is derived by directly comparing the snake models with their backgrounds. This metric is calculated by summing the absolute differences between the targets and backgrounds across each spatial scale. This index is designed to capture variations in both the amplitude and shape of the spectra between patterns. If 2 patterns have similar energy across each granularity scale, this will result in low pattern differences, which reflect as a lower PED, indicative of minimal deviation in amplitude or spectral shape and better background matching (Smithers et al. 2017; Stevens et al. 2017; Price et al. 2019).

#### Color matching

We assessed the degree of luminance and color congruence between the target snakes and their respective backgrounds using a logarithmic form of the receptor noise model (RNL) (Vorobyev and Osorio 1998). This model estimates the color and luminance contrast between the background and the target, interpreted in units of just notice difference (JND). Δ*S* is the discrimination threshold which is determined by the quantum catch and the respective noise level of the receptor channels. Generally, a JND value <1 is considered indistinguishable under ideal viewing conditions, while increasing values above this suggest increasingly discernible differences (Siddiqi et al. 2004; Stevens et al. 2017; Perez-Martinez et al. 2020).

The Weber fractions (*ω*) for evaluating the receptor noise level were calculated by using the color cone density and the Weber fraction of the most abundant cone type (Vorobyev and Osorio 1998). The Weber fraction of red foxes is absent for color discrimination, so we assume a receptor noise of *ω* = 0.05 for color discrimination and used the brightness discrimination in domestic dog as *ω* = 0.22 (Pretterer et al. 2004), and the cone abundance ratio was 34:234 (S:L) according to the retinal photoreceptors of foxes (Malkemper and Peichl 2018). For the black kite model, we assume *ω* = 0.1 and cone abundance ratio of 1:2:2:4 (VS:SWS:MWS:LWS) according to Lind et al. (2013).

Additionally, acuity-corrected (Caves and Johnsen 2018) views with distance scaling were applied in the image analysis to account for the distance-dependent blur, using the following visual acuities: red fox at 8.7 cycles per degree (cpd) (Malkemper and Peichl 2018), and black kite at 37.3 cpd (Potier et al. 2016). Distance modeling ranged from 1 to 5 m in 1-m increments, with 0.5 m as the lower observation limit for fox vision, and from 2.5 to 15 m in 2.5-m increments for black kite vision.

#### Disruptive coloration

We investigated whether patterns exhibit disruptive coloration and how this effect varies with distance. We used Gabor filtering to quantify achromatic edge disruption, which measures the proportion of false edges orthogonal to the animal's true outline (Troscianko et al. 2017). Since luminance contrast and edge orientation changes are often the main factors affecting disruptive coloration, our analysis focused only on the luminance channel. The GabRat sigma value was set at 3, and both previously mentioned visual systems were employed to model the edge disruption. Distance scaling also applied when performing the GabRat analysis. A higher GabRat value indicates more pronounced edge disruption, and a GabRat value below 0.2 indicates low-level edge disruption (Troscianko et al. 2017; Price et al. 2019).

### Statistical analysis

Initial examination of the PED, and Δ*S* values revealed a positive skew, prompting the application of natural log transformation on PED and square root transformation on Δ*S*s for normalization.

A linear mixed model (LMM) fitted with restricted maximum likelihood was chosen to analyze all experimental factors for each visual system separately. Image metrics served as the response variables in interaction models, including chromatic distance (Δ*S*), pattern energy (PED), and edge disruption (GabRat). Fixed effects in the models included pattern, habitat type, viewing angle, and their interactions, acknowledging the distinct characteristics of patterns and habitats under different viewing angles. Body color and viewing distance were also treated as fixed effects, without considering interactions. Point ID was included as a random effect, mitigating the influence of point selection. ANOVAs were used to determine the significance of effects or interactions on response variables, followed by Tukey post hoc tests for within-effect level comparisons. All statistical tests were conducted in R v4.2.3 (R Core Team 2021) using the lme4 package v1.1-35.1 (Bates et al. 2015).

## Results

**Figure 2:**
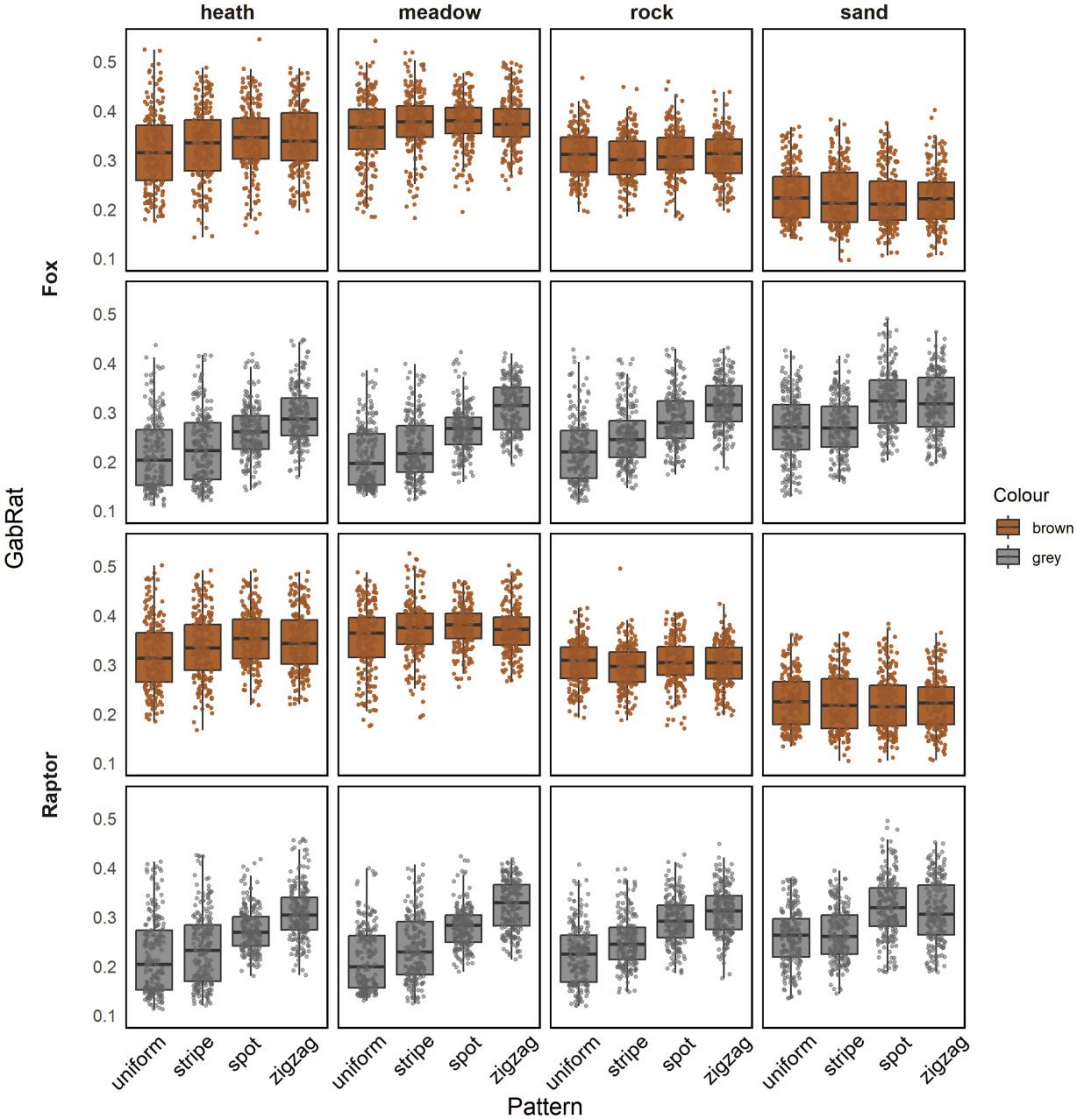
The disruptive value (GabRat) comparison between 4 kinds of snake pattern for all the body colour and habitat combinations for fox and kite visual systems. The box plots show the median and 25th and 75th percentiles; the whiskers indicate the values within 1.5 times the interquartile range.

For disruptive coloration, measured via GabRat, patterns had a significant effect on edge disruption. Snake models with zigzag (Z) patterns consistently had the highest level of edge disruption compared with other patterns, followed by spot (S) patterns, then striped (L) and lastly uniform (U) patterns (Tables 1 and 2). However, when comparing edge disruption between our different pattern, color and background treatments, zigzag and spot patterns were only significantly more disruptive for brown bodied snakes against the heathland and meadow backgrounds (LMM post hoc, heathland: *t*_U-S_ = −5.576, *P* < 0.001, *t*_U-Z_ = −4.650, *P* < 0.001; meadow: *t*_U-S_ = −4.836, *P* < 0.001, *t*_U-Z_ = −4.011, *P* < 0.001), while for the lighter gray bodied snakes they were more disruptive for all backgrounds barring sand (LMM post hoc, *t*_S-Z_ = 1.400, *P* = 0.500) (Fig. 2, Tables 2 and S1).

**Table 1:** ANOVA and pairwise comparison results for all camouflage metrics in black kite vision.

Group	Factor	Camouflage metrics		
		Δ*S*	PED	GabRat
Pattern	ANOVA	*F*_3,6810_ = 36.008, *P* < 0.001	*F*_3,1055_ = 1.740, *P* = 0.157	*F*_3,6810_ = 282.539, *P* < 0.001
	Stripe—uniform	*t* = −5.473, *P* < 0.001	*t* = −1.774, *P* = 0.287	*t* = 6.412, *P* < 0.001
	Stripe—spot	*t* = 1.661, *P* = 0.345	*t* = −0.680, *P* = 0.905	*t* = −14.158, *P* < 0.001
	Stripe—zigzag	*t* = 4.633, *P* < 0.001	*t* = 0.521, *P* = 0.954	*t* = −19.003, *P* < 0.001
	Uniform—spot	*t* = 7.134, *P* < 0.001	*t* = 1.094, *P* = 0.693	*t* = −20.571, *P* < 0.001
	Uniform—zigzag	*t* = 10.106, *P* < 0.001	*t* = 2.057, *P* = 0.168	*t* = −25.415, *P* < 0.001
	Spot—zigzag	*t* = 2.972, *P* = 0.016	*t* = 1.110, *P* = 0.684	*t* = −4.844, *P* < 0.001
Habitat	ANOVA	*F*_3,68_ = 96.080, *P* < 0.001	*F*_3,68.41_ = 9.543, *P* < 0.001	*F*_3,68_ = 16.965, *P* < 0.001
Angle	ANOVA	*F*_1,6810_ = 711.862, *P* < 0.001	*F*_1,1050_ = 3.468, *P* = 0.063	*F*_1,6810_ = 952.532, *P* < 0.001
	Above—horizon	*t* = 26.681, *P* < 0.001	*t* = 1.862, *P* = 0.063	*t* = −30.863, *P* < 0.001
Color	ANOVA	*F*_1,6810_ = 20,739.464, *P* < 0.001	*F*_1,1050_ = 40.010, *P* < 0.001	*F*_1,6810_ = 808.810, *P* < 0.001
Distance	ANOVA	*F*_1,6810_ = 196.290, *P* < 0.001	…	*F*_1,6810_ = 53.892, *P* < 0.001
Point	ANOVA	LRT = 3,080.8, *P* < 0.001	LRT = 624.52, *P* < 0.001	LRT = 1,081.1, *P* < 0.001
Interaction	Pattern:habitat	*F*_9,6810_ = 19.161, *P* < 0.001	*F*_9,1055_ = 0.615, *P* = 0.785	*F*_9,6810_ = 8.303, *P* < 0.001
	Pattern:angle	*F*_3,6810_ = 0.404, *P* = 0.750	*F*_3,1050_ = 0.381, *P* = 0.767	*F*_3,6810_ = 106.637, *P* < 0.001
	Habitat:angle	*F*_3,6810_ = 377.235, *P* < 0.001	*F*_3,1050_ = 10.648, *P* < 0.001	*F*_3,6810_ = 225.270, *P* < 0.001

**Table 2:** ANOVA and pairwise comparison results for all camouflage metrics in red fox vision.

Group	Factor	Camouflage metrics		
		Δ*S*	PED	GabRat
Pattern	ANOVA	*F*_3,6810_ = 99.590, *P* < 0.001	*F*_3,1055_ = 1.847, *P* = 0.137	*F*_3,6810_ = 202.974, *P* < 0.001
	Stripe—uniform	*t* = 5.270, *P* < 0.001	*t* = −1.853, *P* = 0.249	*t* = 5.084, *P* < 0.001
	Stripe—spot	*t* = −0.827, *P* = 0.842	*t* = −0.727, *P* = 0.887	*t* = −11.125, *P* < 0.001
	Stripe—zigzag	*t* = −11.557, *P* < 0.001	*t* = 0.496, *P* = 0.960	*t* = −16.936, *P* < 0.001
	Uniform—spot	*t* = −6.097, *P* < 0.001	*t* = 1.127, *P* = 0.673	*t* = −16.209, *P* < 0.001
	Uniform—zigzag	*t* = −16.828, *P* < 0.001	*t* = 2.101, *P* = 0.153	*t* = −22.020, *P* < 0.001
	Spot—zigzag	*t* = −10.731, *P* < 0.001	*t* = 1.126, *P* = 0.674	*t* = −5.811, *P* < 0.001
Habitat	ANOVA	*F*_3,68_ = 10.828, *P* < 0.001	*F*_3,69_ = 11.099, *P* < 0.001	*F*_3,69_ = 11.664, *P* < 0.001
Angle	ANOVA	*F*_1,6810_ = 126.639, *P* < 0.001	*F*_1,1050_ = 2.294, *P* = 0.130	*F*_1,6810_ = 982.449, *P* < 0.001
	Above—horizon	*t* = 11.253, *P* < 0.001	*t* = 1.515, *P* = 0.130	*t* = −31.344, *P* < 0.001
Color	ANOVA	*F*_1,6810_ = 2,135.007, *P* < 0.001	*F*_1,1050_ = 51.727, *P* < 0.001	*F*_1,6810_ = 919.506, *P* < 0.001
Distance	ANOVA	*F*_1,6810_ = 142.324, *P* < 0.001	…	*F*_1,6810_ = 51.818, *P* < 0.001
Point	ANOVA	LRT = 6,886.9, *P* < 0.001	LRT = 613.85, *P* < 0.001	LRT = 937.38, *P* < 0.001
Interaction	Pattern:habitat	*F*_9,6810_ = 1.964, *P* = 0.127	*F*_9.1055_ = 0.719, *P* = 0.692	*F*_9,6810_ = 5.487, *P* < 0.001
	Pattern:angle	*F*_3,6810_ = 19.413, *P* < 0.001	*F*_3,1050_ = 0.421, *P* = 0.738	*F*_3,6810_ = 65.899, *P* < 0.001
	Habitat:angle	*F*_3,6810_ = 142.135, *P* < 0.001	*F*_3,1050_ = 10.580, *P* < 0.001	*F*_3,6810_ = 179.767, *P* < 0.001

**Figure 3:**
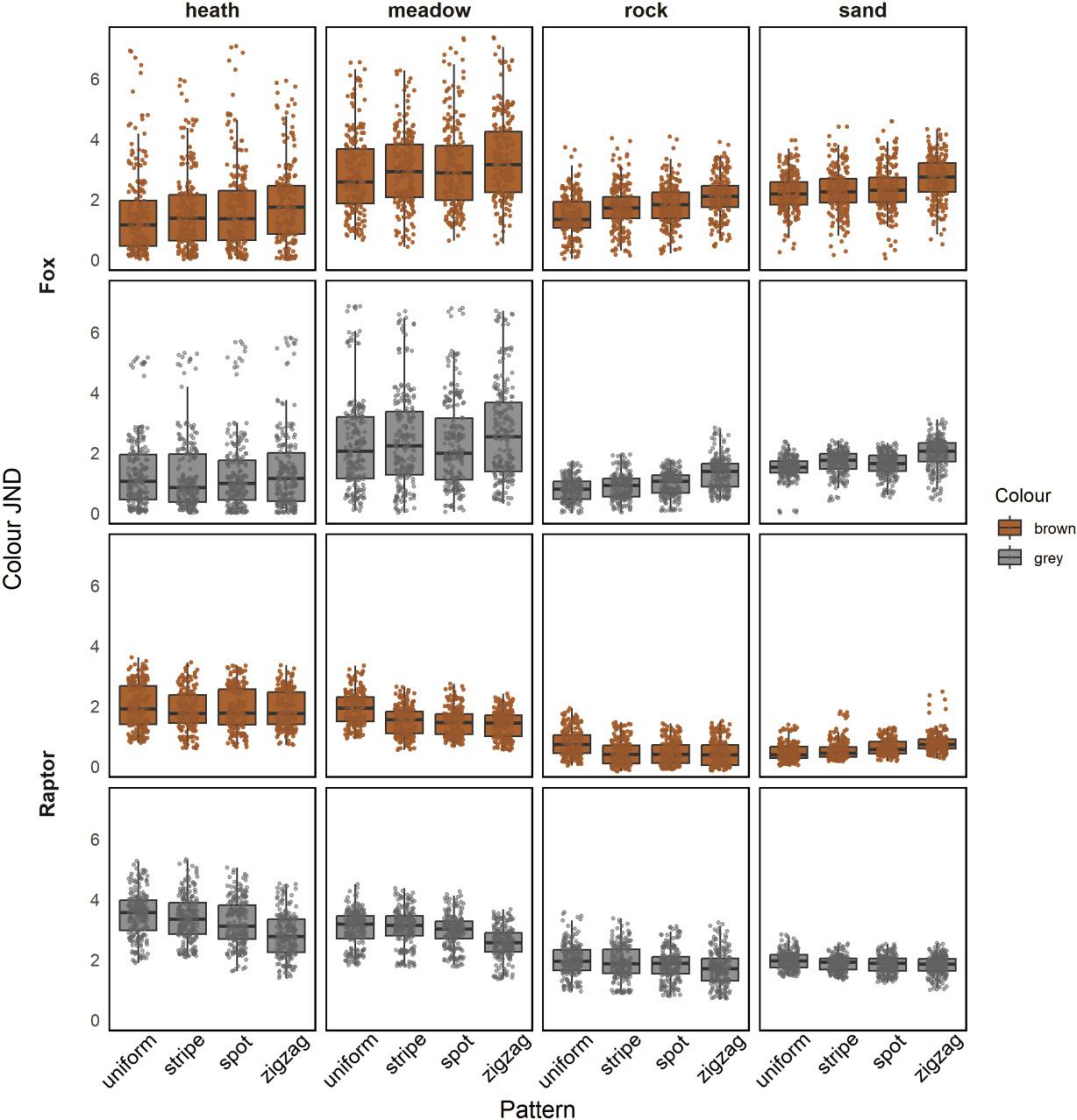
Box plots of chromatic contrast (Δ*S*) comparison in all the body colour and habitat combinations, showing the median and 25th and 75th percentiles; the whiskers indicate the values within 1.5 times the interquartile range. The modeled acuity for black vision was 37.3 cpd and fox was 8.7 cpd.

The effect of pattern on chromatic contrast relative to the background (Δ*S*) varied for different types of visual system. For the red fox's vision, the patterns increased the color difference against the background (Table 1), while for black kite's vision patterns reduced color difference (Table 2). There was no difference between the spot and line pattern types, and the zigzag pattern produced a larger increase in chromatic distance than line or spot in the fox visual model, and the largest reduction in the raptor visual model (Fig. 3, Tables 1 and 2). The pattern effect was also influenced by the habitat type. For fox vision, patterns only increased color contrast from the background in more open and unshaded rocky and sandy habitats (Fig. 3, Table S2). For raptors, brown targets were closer to the background color than gray, regardless of pattern type, and were difficult to detect in open backgrounds (Δ*S* < 1). For gray targets, which were more conspicuous than brown, the patterns reduced the color difference from the background only in habitats with more vegetation, and had no effect when located in open backgrounds (Fig. 3, Table S1).

Our examination of pattern energy, measured as PED, revealed that different patterns did not significantly alter the level of pattern background matching. We found no substantial impact of the patterns on the PED values (Tables 1 and 2). The only fixed effects that influenced PED were color and habitat, which indicates that the pattern variation of the snake models does not contribute to the energy change on most spatial frequency scales, nor influence the pattern matching.

### Observer angle's influence on disruptive coloration (GabRat)

**Figure 4:**
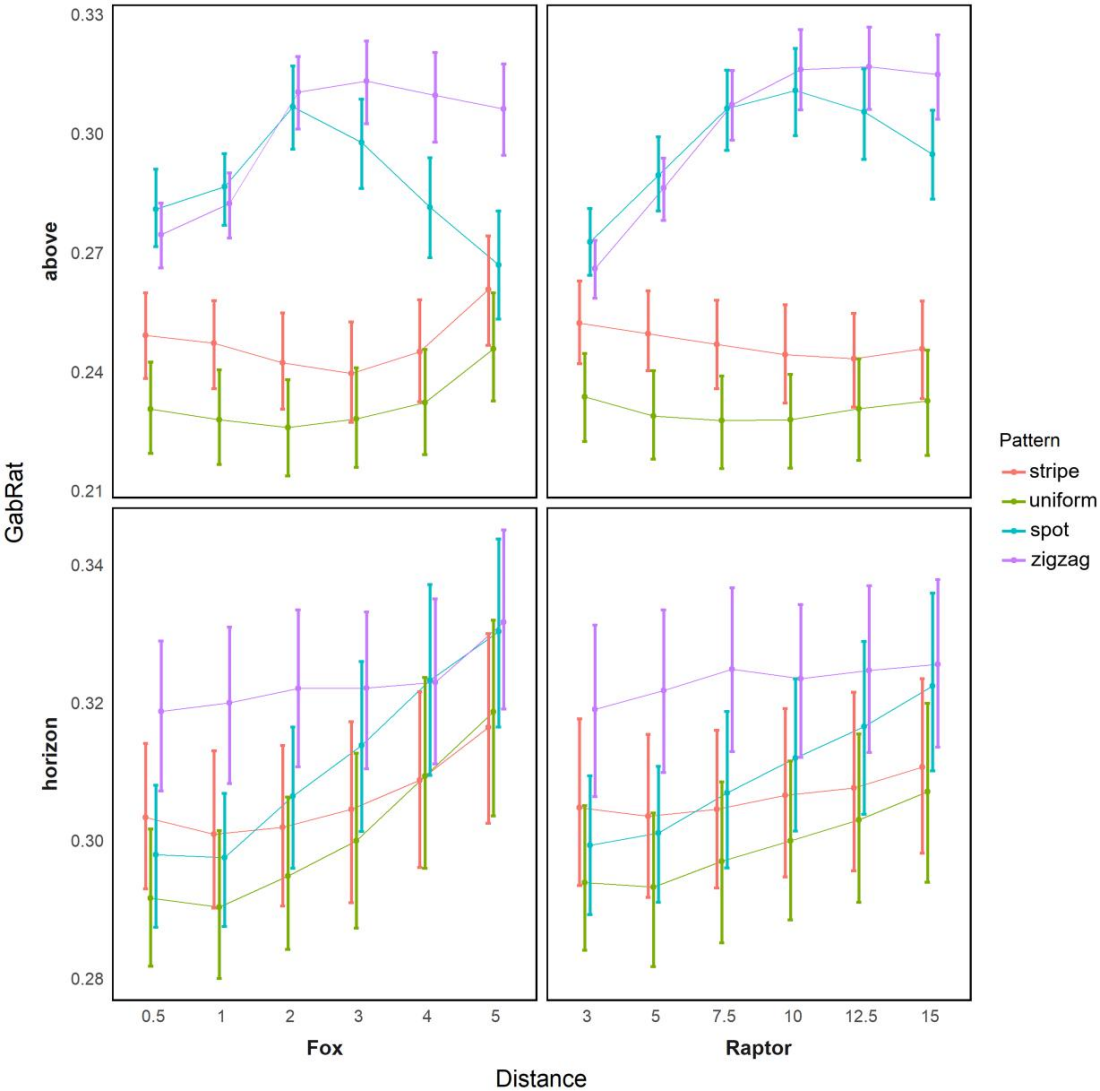
GabRat comparison between 4 kinds of snake pattern at each observation distance and to different visual system, showing the effect of distance on edge disruption. Error bar plot shows the mean and 95% confidence intervals and the central marker indicates the mean value.

Viewing angle significantly affected chromatic background matching (Δ*S*) and disruptive coloration (GabRat), but not pattern background matching (PED) (Tables 1 and 2). For an overhead observer, color difference from the background was higher and the edge disruptive effect was lower compared with a viewing angle of 20 to 30°. The interaction between angle and habitat significantly impacted all metrics, while the interaction between angle and pattern significantly influenced only the GabRat value (Tables 1 and 2). Zigzag patterns consistently enhanced edge disruption compared with other patterns at both heights, whereas spot patterns had a greater effect than stripes when observed from a high angle. And to black kite vision, spots had no significant difference from zigzag markings when viewed from above (Table S3, Fig. 4).

### Effect of patterns influenced by distance

We observed that the impact of spot and zigzag patterns on edge disruption was predominantly from overhead perspectives, but the zigzag pattern showed a greater improvement on edge disruption from longer distances. For these 2 groups, closer observation distances resulted in lower GabRat values. Both pattern groups reached the maximum GabRat value at 2 m to mammalian vision (spot: *t*_1 to 2_ = −10.029, *P* < 0.001, *t*_2 to 3_ = 3.8644, *P* < 0.001; zigzag: *t*_1 to 2_ = −12.302, *P* < 0.001, *t*_2 to 3_ = 1.819, *P* = 0.071) and 10 m to avian vision (spot: *t*_7.5 to 10_ = −3.328, *P* = 0.001, *t*_10 to 12.5_ = 3.713, *P* < 0.001; zigzag: *t*_7.5 to 10_ = −8.283, *P* < 0.001, *t*_10 to 12.5_ = −0.74, *P* = 0.46), indicating that the disruptive effect was highest at this distance. In the spot group, GabRat started to decrease as the distance increased from 3 to 12.5 m to both visual models, and there was no significant change in the zigzag group with distance (Fig. 4). This showed that the zigzag pattern is more effective than the spot pattern at avoiding detection from elevated viewpoints, which is achieved through greater edge disruption when observed from a greater distance; but when the observer is approaching, the detectability increases.

## Discussion

We studied the camouflage capabilities conferred by commonly occurring snake markings and coloration in various habitats. This study enriches existing research which has predominantly focused on the survival advantages these patterns under predation, with limited analysis of the underlying mechanisms (Bittner 2003; Wüster et al. 2004; Valkonen et al. 2011; Valkonen et al. 2020). Our study bridges this gap, and illustrates the specific ways in which these patterns enhance defensive coloration across diverse environments. Notably, our findings show that the snake patterns can have diverse functions which depend on color and contrast of the rest of the body from the background, alongside viewing angle, suggesting a complex evolutionary adaptation for pattern evolution, and underscoring the dynamic interplay between prey and background choice in the natural world.

Compared with snake models with uniform coloration or striped patterns, those with spots and zigzag patterns exhibit higher edge disruption across various habitats, likely due to their intersection with the snake's true edge. This suggests that snakes with such patterns may use camouflage as a primary anti-predator strategy, consistent with the predictions of Allen et al. (2013). By contrast, snakes with stripe patterns may be more likely to rely on anti-predator mechanisms other than camouflage, such as motion dazzle (Stevens et al. 2008; Scott-Samuel et al. 2011; Hämäläinen et al. 2015). Snakes with uniform, lightly speckled, or striped patterns are more likely to employ a flight response rather than staying still to avoid detection compared with spotted snakes, possibly because predators find it difficult to visually track them at high speeds (Brodie 1992). Snake models with longitudinal stripe patterns or conspicuous uniform coloration have been found to be more frequently attacked/detected in multiple experiments comparing with spot or zigzag patterns (Bittner 2003; Wüster et al. 2004; Niskanen and Mappes 2005; Valkonen et al. 2011; Valkonen et al. 2020), which further indicates their incompatibility with effective camouflage when stationary.

Our research demonstrates the benefits of zigzag patterns for camouflage, and that their effects are not uniform across habitats. Many snake species bear a dorsal zigzag pattern and are widely distributed across various habitats; eg, the European adder occurs across habitats including heathland, moorland, grasslands, and woodland edges; viperine water snake (*N. maura*) can be found in swamps and humid meadows; and Lataste's Viper (*Vipera latastei*) tends to occupy deciduous forest and rocky slopes. Previous studies have considered these patterns to have an aposematic function (Speed et al. 2010; Valkonen et al. 2011) or to be related to movement (Stevens et al. 2008; Hämäläinen et al. 2015; Scott-Samuel et al. 2023). Multiple predation experiments using artificial model snakes confirmed an aposematic function of the zigzag patterns, but not a function in camouflage (Wüster et al. 2004; Niskanen and Mappes 2005; Valkonen et al. 2011). However, our findings suggest that a zigzag pattern may decrease the color contrast of the snake against the background when the snake's body color has lower contrast against habitats with more vegetation. By contrast, zigzags increase visibility when snakes are in open habitats and have a higher body color contrast. Such multifunctionality allows zigzag patterns to facilitate adaptation to diverse environments, as evidenced by their wide habitat distribution compared with other patterns (Santos et al. 2018). Santos et al. (2014) supported this conclusion by demonstrating that the size and shape of zigzag patterns vary with the contrast between the snake and its background. In the natural world, some snakes such as adders (Speybroeck et al. 2016), often exhibit both zigzag dorsal patterns and spotted lateral patterns, likely as adaptive traits to accommodate the diverse habitats they inhabit or traverse (Shine and Madsen 1994). When combined with these results, it suggests that the zigzag pattern is likely a habitat-dependent multifunctional antipredator trait.

Crucially, the influence of pattern and habitat were found to change with distance: zigzag patterns disrupt the snake's outline effectively at a distance, but this disruptive effect diminishes when viewed up close. This distance dependence aligns with broader evidence that pattern function can shift with observation distance in other taxa (eg skunk, Caro et al. 2013; poison frog, Barnett et al. 2018; giant panda, Nokelainen et al. 2021) and with the fact that animal eyes vary greatly in their sensitivity to patterns across different distances (Caves et al. 2018). Thus, our results add to the existing evidence that the zigzag patterns of snakes might play a dual role for both camouflage, when viewed at a distance, and signaling when viewed up close (Valkonen et al. 2020). By contrast, the camouflage benefits of stripes and spots is less clear. Spot patterns are likely to afford some degree of camouflage protection, likely through background matching, whereas stripes may have a limited camouflage value. However, adjusting the spacing between and size of the markings could be crucial for remaining inconspicuous to different predators and at different distances. Whether the size of markings should match background features exactly is unclear, with some studies showing that matching increases survival (Barnett et al. 2017b), while others have found that markings smaller then background features enhances survival rates (Barnett et al. 2017a). Nonetheless, it is clear that the cryptic effect of patterns, especially zigzags, is dependent on distance.

The differences in camouflage effectiveness between brown and gray model snakes suggest that interactions between base color and dorsal pattern can yield different outcomes across body colors. These 2 base colors were chosen to represent alternative and commonly occurring color morphs in snakes, including (but not restricted to) vipers in our study region. In some vipers, behavioral records indicate that during the mating season, gray males often undertake long journeys to find receptive females, usually traveling through various habitats, including open areas, while brown females tend to stay in more sheltered locations (Andrén 1985; Shine and Madsen 1994). These differences in behavior and habitat are likely to lead to differing camouflage requirements. Previous studies have found that brown artificial snakes are less likely to be detected by human observers compared with gray ones (Valkonen et al. 2020). Our study partially confirms this viewpoint, showing that brown body color enhances the concealment of the snake model. However, because gray individuals exhibit higher internal luminance contrast between the black markings and the lighter base color, gray snakes can also benefit from a zigzag pattern, by reducing detectability in sheltered habitats, and increasing detectability in open habitats to potentially signal their defenses (aposematism). Snakes in open habitats may stand out against the background, making them more susceptible to predation (de Avila et al. 2019; Harmel et al. 2020). Our results suggest that brown snakes derive limited benefit from zigzag patterns, as their body color alone provides sufficient camouflage or conspicuousness depending on the context. This indicates that among snakes with multiple color morphs, perhaps lighter individuals benefit more from zigzag patterns, while darker individuals rely more on their body color for background matching. Clearly, the advantages of zigzag patterns are unbalanced among snakes of different colors, which may help explain the sexual dichromatism in snakes. However, we did not attempt to match the coloration of real snakes to the substrate, which is likely to play an important role in general concealment alongside specific patterns. Additionally, our models did not incorporate UV reflectance, which is now known to be widespread in snakes, that could potentially function in camouflage in some contexts (Crowell et al. 2024). Future research should consider these factors to more comprehensively test the camouflage function of snake patterns.

Previous research has seldom discussed how the angle of observation affects camouflage. However, our study has shown that visual angle significantly affects both background matching and edge disruption to mammal and avian predator visual systems (represented by fox and black kite). In general, higher viewing angles tend to weaken camouflage, whereas lower angles can reduce detectability through environmental occlusion such as vegetation, consistent with Hancock et al.'s (2023) finding that bird nests at lower angles are more obscured to mammalian predators. Regarding different patterns, previous predation experiments have confirmed the effectiveness of zigzag pattern against avian predators (Wüster et al. 2004; Niskanen and Mappes 2005; Valkonen et al. 2011). In our research, changes in dorsal patterns mainly affect edge disruption at various angles, and zigzag markings seem consistently the best camouflage against both avian and mammalian predators regardless of the viewing angle. Compared with other pattern types, zigzags maintain contact with the body's outline across a wider range of angles, effectively disrupting the target's true outline (Cott 1940; Stevens et al. 2006; Stevens and Merilaita 2009). However, considering putative disruptive patterns (spots, zigzag) strengthen camouflage more effectively than other patterns when observed from above than lower angles, the zigzag pattern likely evolved to counter aerial predators.

## Conclusion

Our study highlights the diverse antipredator functions of common snake patterns, demonstrating their ability to facilitate both camouflage and aposematism depending on the ecological context. Zigzag and spot patterns were particularly effective in disrupting the snakes’ outline, enhancing crypsis at a distance, whereas stripe patterns offered less concealment, suggesting alternative functions such as motion dazzle. Moreover, the impact of these patterns varied across different habitats, with open environments amplifying the aposematic potential of zigzag markings, whereas vegetated backgrounds favored cryptic strategies. Viewing angle played a crucial role in camouflage effectiveness, suggesting that pattern functions may alter when perspectives change. These findings underscore the importance of considering multiple ecological and perceptual factors when studying animal coloration. Rather than serving a singular function, snake patterns appear to operate flexibly depending on body coloration, viewing conditions, and predator perspective. This multifunctionality likely contributes to the widespread occurrence of certain markings across diverse snake species and habitats. Future research should further investigate real snake populations to validate these findings, examining how evolutionary pressures drive patterns diversity and behavioral adaptations. Additionally, more camouflage studies should consider photographing animals/models at multiple viewing angles relevant to natural observers.

## Supplementary Material

arag051_Supplementary_Data

## Data Availability

Analyses reported in this article can be reproduced using the data provided by Li (2026).
