# Supplementary material for: Snake markings facilitate diverse anti-predator functions depending on habitat and viewing angle
Source: Behav Ecol. 2026 May 18;37(4):arag051. doi: 10.1093/beheco/arag051 (PMC13202211; doi:10.1093/beheco/arag051)
Supplement: arag051_Supplementary_Data [file arag051_supplementary_data.docx]

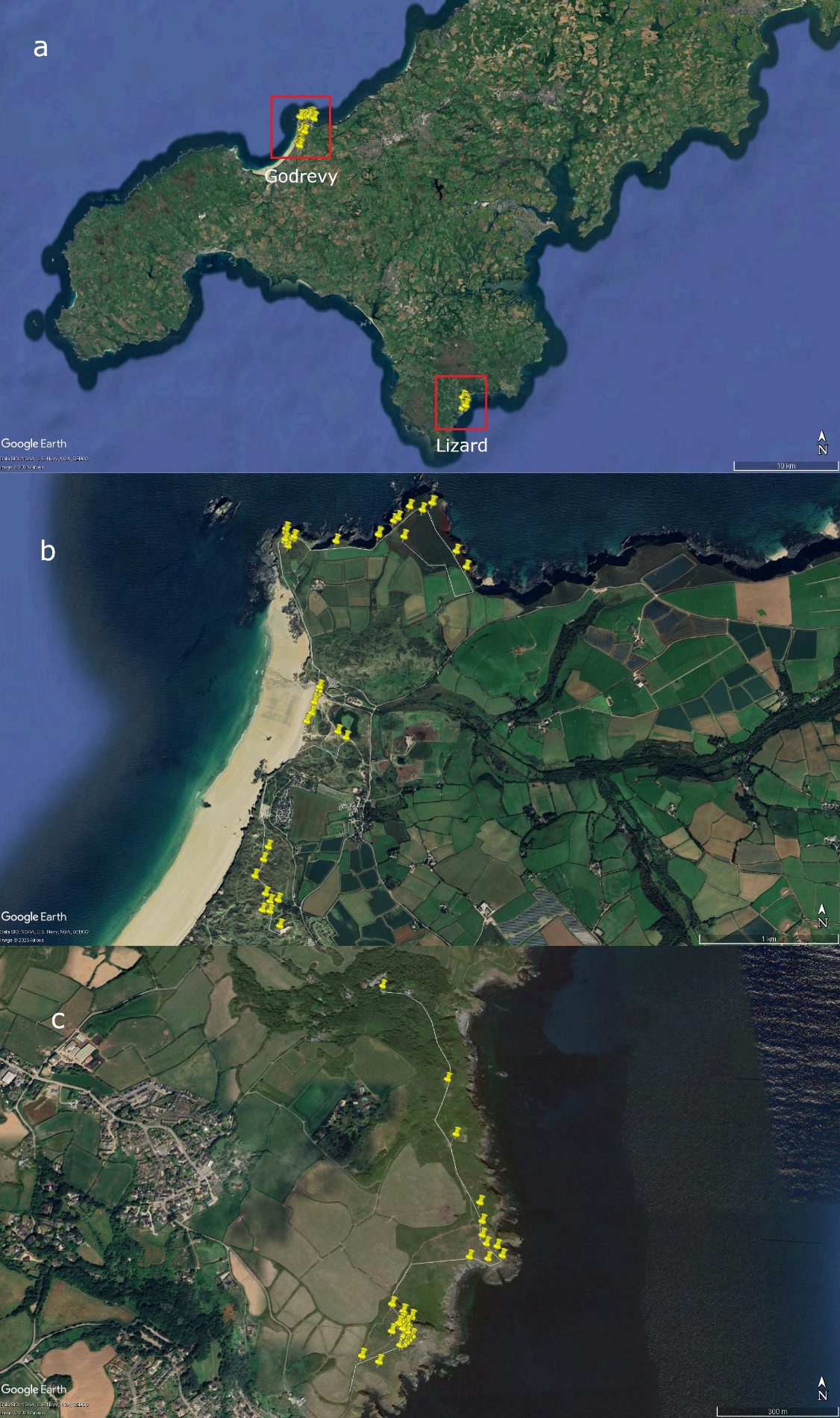


Figure S1. The map of transect and site selection. a. map of Cornwall area, the red square marked the location of the reserves. b. the map shows the transect and site in Godrevy area. c. the map shows the transect and site in Lizard area.

| **Table S1. Pairwise comparison of patterns in colour-habitat pairs for camouflage metrics in black kite vision.** | | | | | | |
| --- | --- | --- | --- | --- | --- | --- |
| metric | colour | level | Habitat | | | |
|  |  |  | Heathland | Meadow | Rocky slope | Sands |
| GabRat | Brown | stripe - uniform | t=2.765, p=0.030 | t=3.660, p=0.002 | t=-2.074, p=0.163 | t=-0.391, p=0.980 |
|  |  | stripe - spot | t=-2.811, p=0.126 | t=-1.176, p=0.642 | t=-2.762, p=0.030 | t=0.336, p=0.987 |
|  |  | stripe - zigzag | t=-1.885, p=0.235 | t=-0.351, p=0.985 | t=-1.631, p=0.362 | t=0.596, p=0.933 |
|  |  | uniform - spot | t=-5.576, p<0.001 | t=-4.836, p<0.001 | t=-0.688, p=0.902 | t=0.726, p=0.887 |
|  |  | uniform - zigzag | t=-4.650, p<0.001 | t=-4.011, p<0.001 | t=0.443, p=0.971 | t=0.987, p=0.757 |
|  |  | spot - zigzag | t=0.925, p=0.791 | t=0.824, p=0.843 | t=1.131, p=0.670 | t=0.260, p=0.994 |
|  | Grey | stripe - uniform | t=2.211, p=0.121 | t=4.149, p<0.001 | t=5.748, p<0.001 | t=1.041, p=0.726 |
|  |  | stripe - spot | t=-6.477, p<0.001 | t=-8.034, p<0.001 | t=-8.257, p<0.001 | t=-9.476, p<0.001 |
|  |  | stripe - zigzag | t=-11.94, p<0.001 | t=-16.11, p<0.001 | t=-12.160, p<0.001 | t=-8.076, p<0.001 |
|  |  | uniform - spot | t=-8.688, p<0.001 | t=-12.182, p<0.001 | t=-14.005, p<0.001 | t=-10.517, p<0.001 |
|  |  | uniform - zigzag | t=-14.151, p<0.001 | t=-20.259, p<0.001 | t=-17.908, p<0.001 | t=-9.117, p<0.001 |
|  |  | spot - zigzag | t=-5.463, p<0.001 | t=-8.076, p<0.001 | t=-3.903, p=0.001 | t=1.400, p=0.500 |
| ΔS | Brown | stripe - uniform | t=-2.100, p=0.154 | t=-4.359, p<0.001 | t=-2.988, p=0.015 | t=2.375, p=0.083 |
|  |  | uniform - spot | t=1.176, p=0.642 | t=5.409, p<0.001 | t=3.225, p=0.007 | t=-3.723, p=0.001 |
|  |  | uniform - zigzag | t=1.503, p=0.436 | t=6.936, p<0.001 | t=3.620, p=0.002 | t=-8.499, p<0.001 |
|  |  | stripe - spot | t=-0.924, p=0.792 | t=1.050, p=0.720 | t=0.237, p=0.995 | t=-1.348, p=0.532 |
|  |  | stripe - zigzag | t=-0.598, p=0.933 | t=2.577, p=0.050 | t=0.631, p=0.922 | t=-6.124, p<0.001 |
|  |  | spot - zigzag | t=0.326, p=0.988 | t=1.527, p=0.422 | t=0.394, p=0.979 | t=-4.776, p<0.001 |
|  | Grey | stripe - uniform | t=-1.304, p=0.561 | t=0.575, p=0.940 | t=-1.693, p=0.328 | t=-3.387, p=0.004 |
|  |  | uniform - spot | t=2.953, p=0.017 | t=1.237, p=0.604 | t=2.854, p=0.023 | t=4.809, p<0.001 |
|  |  | uniform - zigzag | t=5.808, p<0.001 | t=5.007, p<0.001 | t=4.495, p<0.001 | t=5.373, p<0.001 |
|  |  | stripe - spot | t=1.649, p=0.352 | t=1.812, p=0.268 | t=1.161, p=0.652 | t=1.423, p=0.485 |
|  |  | stripe - zigzag | t=4.504, p<0.001 | t=5.582, p<0.001 | t=2.802, p=0.027 | t=1.986, p=0.194 |
|  |  | spot - zigzag | t=2.855, p=0.023 | t=3.770, p=0.001 | t=1.642, p=0.356 | t=0.563, p=0.943 |

| **Table S2. Pairwise comparison of patterns in colour-habitat pairs for camouflage metrics in red fox vision.** | | | | | | |
| --- | --- | --- | --- | --- | --- | --- |
| metric | colour | level | Habitat | | | |
|  |  |  | Heathland | Meadow | Rocky slope | Sands |
| GabRat | Brown | stripe - uniform | t=2.267, p=0.107 | t=2.782, p=0.028 | t=-2.219, p=0.119 | t=-0.752, p=0.876 |
|  |  | stripe - spot | t=-1.621, p=0.367 | t=-0.366, p=0.983 | t=-1.816, p=0.266 | t=0.507, p=0.958 |
|  |  | stripe - zigzag | t=-1.601, p=0.379 | t=-0.2, p=0.997 | t=-1.289, p=0.570 | t=0.550, p=0.947 |
|  |  | uniform - spot | t=-3.888, p<0.001 | t=-3.149, p=0.009 | t=0.403, p=0.978 | t=1.258, p=0.590 |
|  |  | uniform - zigzag | t=-3.868, p<0.001 | t=-2.982, p=0.016 | t=0.930, p=0.789 | t=1.301, p=0.562 |
|  |  | spot - zigzag | t=0.020, p=1.000 | t=0.166, p=0.998 | t=0.527, p=0.953 | t=0.043, p=1.000 |
|  | Grey | stripe - uniform | t=2.155, p=0.137 | t=3.588, p=0.002 | t=5.229, p<0.001 | t=0.640, p=0.919 |
|  |  | stripe - spot | t=-5.484, p<0.001 | t=-7.135, p<0.001 | t=-6.146, p<0.001 | t=-9.123, p<0.001 |
|  |  | stripe - zigzag | t=-10.458, p<0.001 | t=-15.218, p<0.001 | t=-11.624, p<0.001 | t=-7.932, p<0.001 |
|  |  | uniform - spot | t=-7.639, p<0.001 | t=-10.723, p<0.001 | t=-11.375, p<0.001 | t=-9.764, p<0.001 |
|  |  | uniform - zigzag | t=-12.613, p<0.001 | t=-18.807, p<0.001 | t=-16.853, p<0.001 | t=-8.572, p<0.001 |
|  |  | spot - zigzag | t=-4.974, p<0.001 | t=-8.084, p<0.001 | t=-5.478, p<0.001 | t=1.191, p=0.633 |
| ΔS | Brown | stripe - uniform | t=0.775, p=0.866 | t=0.774, p=0.866 | t=3.237, p=0.007 | t=0.993, p=0.753 |
|  |  | uniform - spot | t=-1.355, p=0.528 | t=-1.889, p=0.234 | t=-3.294, p=0.006 | t=-2.162, p=0.135 |
|  |  | uniform - zigzag | t=-2.850, p=0.023 | t=-4.097, p<0.001 | t=-5.916, p<0.001 | t=-4.090, p<0.001 |
|  |  | stripe - spot | t=-0.580, p=0.938 | t=-1.114, p=0.681 | t=-0.057, p=1.000 | t=-1.169, p=0.647 |
|  |  | stripe - zigzag | t=-2.075, p=0.162 | t=-3.322, p=0.105 | t=-2.679, p=0.038 | t=-3.097, p=0.011 |
|  |  | spot - zigzag | t=-1.495, p=0.441 | t=-2.208, p=0.122 | t=-2.623, p=0.044 | t=-1.928, p=0.017 |
|  | Grey | stripe - uniform | t=-0.502, p=0.959 | t=1.008, p=0.745 | t=2.481, p=0.064 | t=2.672, p=0.039 |
|  |  | uniform - spot | t=0.216, p=0.996 | t=-0.346, p=0.986 | t=-2.809, p=0.026 | t=-1.308, p=0.558 |
|  |  | uniform - zigzag | t=-1.019, p=0.739 | t=-2.839, p=0.124 | t=-8.038, p<0.001 | t=-6.566, p<0.001 |
|  |  | stripe - spot | t=-0.285, p=0.992 | t=0.662, p=0.911 | t=-0.329, p=0.988 | t=1.364, p=0.523 |
|  |  | stripe - zigzag | t=-1.520, p=0.426 | t=-1.831, p=0.260 | t=-5.558, p<0.001 | t=-3.895, p<0.001 |
|  |  | spot - zigzag | t=-1.235, p=0.605 | t=-2.493, p=0.062 | t=-5.229, p<0.001 | t=-5.258, p<0.001 |

| **Table S3. GabRat pairwise comparison of Angles in two kinds of animal vision.** | | | |
| --- | --- | --- | --- |
| Angle | level | Species | |
|  |  | Black kite | Red fox |
| Above | stripe - uniform | t=5.187, p<0.001 | t=4.510, p<0.001 |
|  | stripe - spot | t=-15.364, p<0.001 | t=-11.521, p<0.001 |
|  | stripe - zigzag | t=-16.790, p<0.001 | t=-15.115, p<0.001 |
|  | uniform - spot | t=-20.552, p<0.001 | t=-16.031, p<0.001 |
|  | uniform - zigzag | t=-21.977, p<0.001 | t=-19.625, p<0.001 |
|  | spot - zigzag | t=-1.426, p=0.483 | t=-3.594, p=0.002 |
| Horizon | stripe - uniform | t=2.097, p=0.154 | t=1.432, p=0.479 |
|  | stripe - spot | t=-0.987, p=0.757 | t=-1.557, p=0.404 |
|  | stripe - zigzag | t=-4.895, p<0.001 | t=-4.684, p<0.001 |
|  | uniform - spot | t=-3.084, p=0.011 | t=-2.989, p=0.015 |
|  | uniform - zigzag | t=-6.993, p<0.001 | t=-6.117, p<0.001 |
|  | spot - zigzag | t=-3.908, p<0.001 | t=-3.128, p=0.010 |
